# Inter- and intraobserver reliability assessment of the axial trunk rotation: manual versus smartphone-aided measurement tools

**DOI:** 10.1186/1471-2474-15-343

**Published:** 2014-10-11

**Authors:** Jun Qiao, Leilei Xu, Zezhang Zhu, Feng Zhu, Zhen Liu, Bangping Qian, Yong Qiu

**Affiliations:** Spine Surgery, Drum Tower Hospital, Nanjing University Medical School, 321 Zhongshan Road, Nanjing, China

**Keywords:** Reliability, Smartphone-aided measurement, Axial trunk rotation, Adolescent idiopathic scoliosis

## Abstract

**Background:**

Scoliogauge, has been developed for the measurement of ATR on iPhone smartphones. This study was to evaluate the reliability for the smartphone-aided ATR measurement method and to compare its reliability with that of the manual method.

**Methods:**

Sixty-four AIS patients with single thoracic or lumbar curve participated in this study. Of these patients, thirty-two patients had main thoracic scoliosis while other thirty-two had main thoracolumbar/lumbar scoliosis. Two spine surgeons performed the measurements with Scoliometer and Scoliogauge. The Scoliogauge measurements were conducted on an iPhone 4 smartphone. The intraclass correlation coefficient (ICC) 2-way mixed model on absolute agreement was used to analyze the reliability categorized according to regions: thoracic or lumbar, and Cobb angles: <20 degrees and >40 degrees. ICC < 0.40 is considered as poor, 0.40–0.59 as fair, 0.60–0.74 as good, and 0.75–1.00 as excellent.

**Results:**

The overall intraobserver variability was 0.954 and the overall interobserver variability was 0.943 for the scoliometer set, whereas the intraobserver variability was 0.965 and interobserver variability was 0.964 for the scoliogauge set. Both the intraobserver and interobserver ICCs reached the excellent value in the 2 sets for both observers. The mean Cobb angle of thoracic curves in patients with main thoracic scoliosis was similar to that of lumbar curves in those with main thoracolumbar/lumbar scoliosis (35.7 degrees vs. 36.1 degrees). The intraobserver and interobserver reliability was similar between two groups (thoracic vs. lumbar) in the 2 sets. There were 21 patients having Cobb angles < 20 degrees, while 20 patients >40 degrees. The intraobserver and interobserver reliability was better in severe curve(>40 degrees) group.

**Conclusion:**

Smartphone-aided measurement for ATR showed excellent reliability, and the reliability of measurement with either scoliometer or scoliogauge could be influenced by Cobb angle that reliability was better for curves with larger Cobb angles.

**Electronic supplementary material:**

The online version of this article (doi:10.1186/1471-2474-15-343) contains supplementary material, which is available to authorized users.

## Background

Adolescent idiopathic scoliosis (AIS) is the most commonly seen spinal deformity that mainly occurs in girls at the peri-pubertal period, and may progress rapidly to significant cosmetic problems and functional disabilities [1–3]. School screening was advocated with the aim of early diagnosis, so that conservative treatments could be adopted timely and subsequently minimizes the possibility of surgical treatment [4, 5]. Scoliometer, a portable inclinometer-based device, which has been widely used in school screening, has showed satisfactory specificity and sensitivity [6–8]. Moreover, Scoliometer is a good noninvasive modality monitoring the progression of AIS without the concern of radiation exposure to patients [9, 10]. Nevertheless, the relatively high price, as high as 89 dollars at Amazon online store, may hinder.

The popularization of this instrument. Furthermore, this device is not available in every region especially in developing country. Fortunately, the development of smartphones seems to address these problems. An accelerometer-based application (app), Scoliogauge, has been developed for the measurement of ATR on iPhone smartphones, with a price of only 0.99 dollar. Physicians and patients who have iPhones could download this app from online-store at any place. This instrument seems to be a good substitute for scoliometer. However, so far there is no evidence available to prove the reliability of this app. It really makes sense to assess the reliability of this smartphone-aided electrical measurement as compared with traditional Scoliometer before it could be widely accepted. Hence, we performed a comparative study with Scoliometer to investigate the reliability of this app.

## Methods

**Figure 1:**
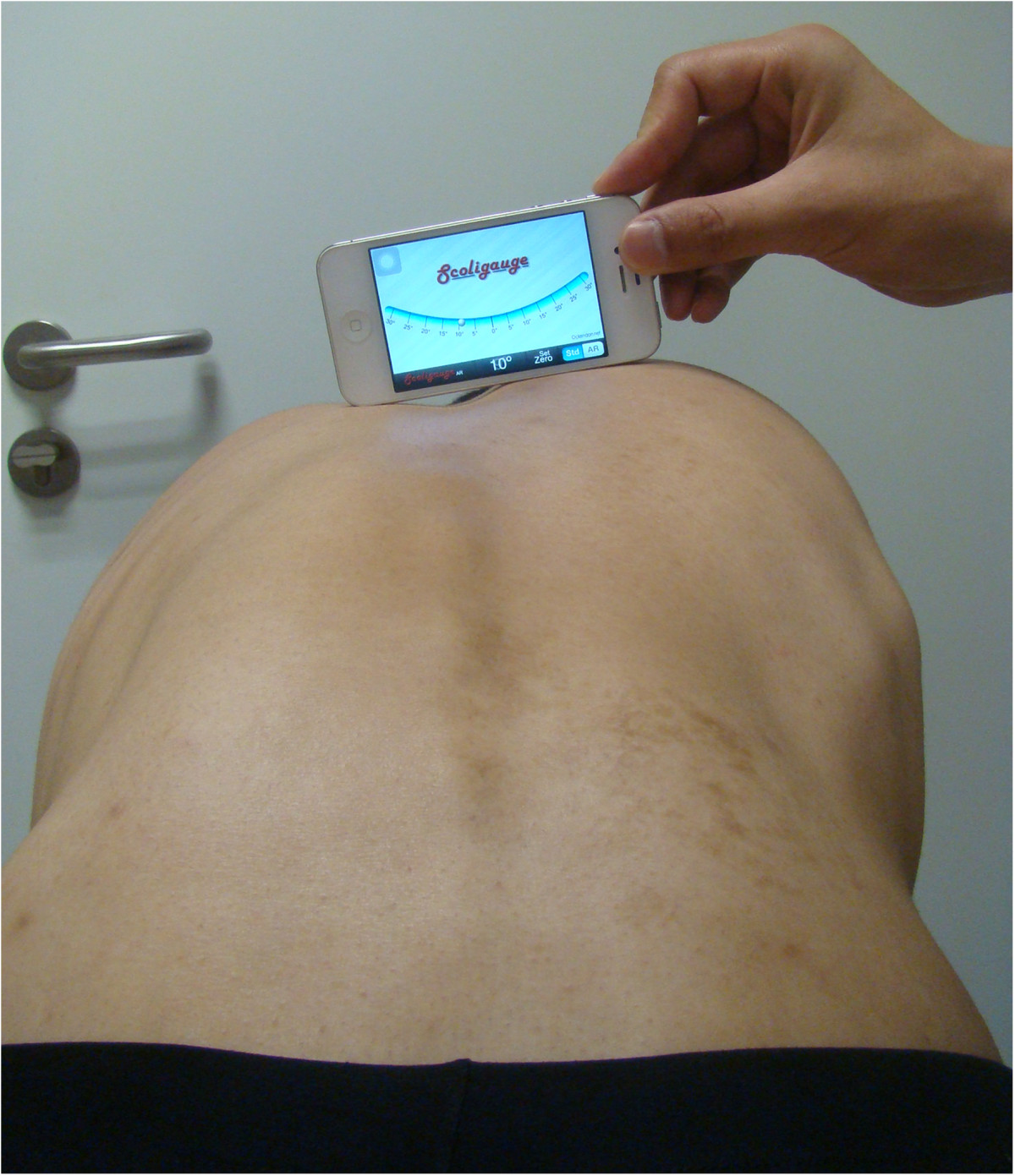
**Illustration of measuring axial trunk rotation (ATR) by Scoliogauge.**

Sixty-four AIS patients with single thoracic or lumbar curve participated in this study. Of these patients, thirty-two patients had main thoracic scoliosis while other thirty-two had main thoracolumbar/lumbar scoliosis. The mean age of the patients was 15.7 years (range, 12 to 16 years). Posteroanterior radiographs were obtained to measure the Cobb angles of scoliosis. All subjects signed the consent form. The approval of the study protocol was granted by the Committee of Ethics of Nanjing University Drum Tower Hospital.Two spine surgeons performed the Scoliometer measurements and Scoliogauge measurements. The Scoliogauge measurements were conducted on an iPhone 4 smartphone. To familiarize the examiners with measurements, certain training program was rendered for practicing, which consisted of measuring 20 patients with main thoracic scoliosis and 20 with main thoracolumbar/lumbar scoliosis using both Scoliometer and Scoliogauge app on a smartphone (Figure 1). These data were not included in statistical analysis. The measurements were performed independently and by convenience, with each examiner being blinded to the other’s measurements. To minimize measurement error, the examiners used the same instrument and smartphone to assess all patients.

During the measurement, all subjects were barefoot. Female participants had their hair tied up and were using a specific shirt that allowed the exposure of the entire back while male subjects were topless. The examiners placed the Scoliometer or smartphone on the thoracic or lumbar spine while the patient performed Adam’s forward bend test. When performing smartphone measurement, the examiners placed their thumbs between the patient and either end of the device to accommodate prominent spinous process. The patients stood with their feet shoulder-width apart, held the hands together with uplimbs in front their body. Depending on the location of the thoracic rotational prominence or lumbar flank prominence, the patients was asked to bend forward 90 degree until the hump become very apparent. The examiners placed the Scoliometer or smartphone at the siteof the most prominent partof the hump, with the “0” mark centered over the spine. Scoliometer and Scoliogauge measurements were performedinthe thoracic regions for patients with thoracic scoliosis and lumbar regions for those with thoracolumbar/lumbar scoliosis. Each examiner performed two evaluations. There were 15-20 minutes of interval between the first and second evaluation of one examiner.

### Statistical methods

The intraclass correlation coefficient (ICC) 2-way mixed model on absolute agreement was used to analyze measurement reliability in categories according to regions: thoracic or lumbar, and Cobb angles: <20 degrees and >40 degrees. The values of the ICC may range from 0 to 1, with a higher value indicating better reliability. ICC < 0.40 is considered as poor, 0.40–0.59 as fair, 0.60–0.74 as good, and 0.75–1.00 as excellent. Statistical analyses were performed using SPSS 13.0 software (SPSS Inc., Chicago, IL).

## Results

The overall intraobserver variability was 0.954 and the overall interobserver variability was 0.943 for the scoliometer set, whereas the intraobserver variability was 0.965 and interobserver variability was 0.964 for the scoliogauge set. Both the intraobserver and interobserver ICCs were excellent in the 2 sets for both 2 observers (Table 1).

**Table 1:** **Intraclass correlation coefficients (ICC) in Scoliometer and scoliogauge measurement**

	Scoliometer					Scoliogauge				
	All (n = 64)	Thoracic (n = 32)	Lumbar (n = 32)	Large Cobb (n = 20)	Small Cobb (n = 21)	All (n = 64)	Thoracic (n = 32)	Lumbar (n = 32)	Large Cobb (n = 20)	Small Cobb (n = 21)
Intraobserver 1	0.960	0.958	0.963	0.976	0.782	0.959	0.961	0.963	0.973	0.772
Intraobserver 2	0.953	0.951	0.960	0.967	0.741	0.967	0.965	0.968	0.972	0.759
Overall intraobserver	0.954	0.952	0.961	0.972	0.758	0.965	0.963	0.964	0.973	0.764
Interobserver	0.943	0.939	0.948	0.976	0.821	0.964	0.965	0.972	0.971	0.819

The mean Cobb angle of thoracic curves in patients with main thoracic scoliosis was similar to that of lumbar curves in those with main thoracolumbar/lumbar scoliosis (35.7 degrees vs. 36.1 degrees). The intraobserver and interobserver reliability was similar between two groups (thoracic vs. lumbar) in the 2 sets for all 2 observers. There were 21 patients having Cobb angles < 20 degrees, while 20 patients > 40 degrees. The intraobserver and interobserver reliability was better in larger Cobb angle (>40 degrees) group (Table 1).

## Discussion

The popularity of smartphones has provided new opportunities that integrate mobile technology into daily clinical practices. A recent study surveying the use of smartphones among orthopedic surgeons, demonstrated that 84% of respondents have a smartphone, the majority (55%) have an iPhone, and that 53% of people with smart- phones already use applications in clinical practice [11]. Several apps have been developed to assist the diagnosis and treatment of orthopedic disease. As to scoliosis, there are two apps, CobbMeter and Scoliogauge. CobbMeter is a smartphone application aimed to rapidly measure the Cobb angles for scoliosis on iPhone smartphones. With the assistance of CobbMeter application, surgeons can perform measurements on either hard copies or digital radiographs. A reliability analysis has been performed to evaluate the consistency and measurement error of this smartphone-aided Cobb angle measurement method and compare its reliable characteristics with those of the manual method, demonstrating thatboth the intraobserver ICC and the interobserver ICC were better in the smartphone set than in the manual set [12]. In clinical practices, we have widely used CobbMeter to measure Cobb angle. As another frequently used instrument for scoliosis, Scoliogauge had not been investigated in terms of its reliability. So we performed this study to lay the foundation of the widespread use of this smartphone-based instrument.

The reliability and validity of Scoliometer have been widely studied. Amendt et al. conducted the first reliability analysis of Scoliometer measuring ATR in individuals with scoliosis, and reported that the intra-rater and inter-rater reliability coefficients of Scoliometer were high (r = 0.86-0.97) showing good measurement reproducibility [6]. Cote et al. performed a more comprehensive study that stratified the results by regions demonstrating inter-rater reliability of 0.91 for the thoracic region and 0.74 for the lumbar region [13]. In a recent study, Bonagamba et al. measured ATR at each level of spine, from T1 to L5, and indicated that both intra- and inter- rater reliability were lowest at upper thoracic region (T1 to T4) and highest at lower thoracic region (T9 to T12). The author ascribed the low reliability to the effect of cervical rotation on the measurement that any movement of cervical spine would change the shape of thoracic region of spine, thus introducing bias in evaluation [8]. In the present study, the intra- and inter-rater reliability was similar between Scoliometer measurement and Scoliogauge measurement. In theory, the sources of variability of the measurement performed with either the scoliometer or scoliogauge comes from the process of positioning, palpation and determination of the spinous process. For the instruments themselves, there should be no difference. We also compared the intra- and inter-rater reliability of measurements between thoracic region and lumbar region, and found similar results between two regions, which was nonconsistent with most of the previous studies. We suspected that the difference of reliability among different regions might be due to different Cobb angles of curves in these regions. Most of these studies included patients with primary thoracic curve, which was larger than lumbar curve [6, 14]. As larger Cobb angles, more prominent hump, easier identification of measurement site, it is not surprising to get better reliability on thoracic region in these studies. Only one study included this factor into considerations that recruited patients with single thoracic curves and double major curves demonstrating that the reliabilities were similar between thoracic and lumbar regions for double major curves, and better at thoracic region for single thoracic curves [15]. In our study, two groups, thoracic and lumbar, had similar Cobb angles that eliminated the effect of Cobb angle on the reliability analysis. In order to prove our postulation, we also stratified the results by Cobb angle (<20 degrees vs. >40 degrees), finding that the intra- and inter-rater reliability was better in the group with large Cobb angle for both scoliometer and scoliogauge.

The present study laid the foundation of the widespread use of this smartphone-based instrument for the measurement of ATR of scoliosis, and first demonstrated the influential factor for the reliability of the measurement of ATR. The most significant limitation of this study is the relatively small volume of subjects. Moreover, the measurement of ATR by Scoliogauge was not compared with that in CT scans, which is the most accurate method for the measurement of ATR. However, the main aim of this study was to investigate the reliability of this state of art instrument versus that of traditional manual measurement instrument, as the strong correlation between this back surface measurement method and radiographic measurement method had been verified in previous studies [16, 17].

## Conclusions

In conclusion, smartphone-aided measurement for ATR showed excellent reliability, and the reliability of measurement by either scoliometer or scoliogauge was influenced by Cobb angle that reliability was better for curves with larger Cobb angles.

## Consent

Written informed consent was obtained from all patients and their parents enrolled in the investigation. The person in the image has specifically provided consent to publish their image. The study protocol conformed to the ethical guidelines of the 1975 Declaration of Helsinki and the guidelines of the regional ethical committees of Zurich, Switzerland, and Basel, Switzerland.

## Authors’ original submitted files for images

Below are the links to the authors’ original submitted files for images. Authors’ original file for figure 1

## Abbreviations

AIS: Adolescent idiopathic scoliosis
ATR: Axial trunk rotation
ICC: Intraclass correlation coefficient.
