# Supplementary figures and images for: Inter- and intraobserver reliability assessment of the axial trunk rotation: manual versus smartphone-aided measurement tools
Source: BMC Musculoskelet Disord. 2014 Oct 11;15:343. doi: 10.1186/1471-2474-15-343 (PMC4198676; doi:10.1186/1471-2474-15-343)

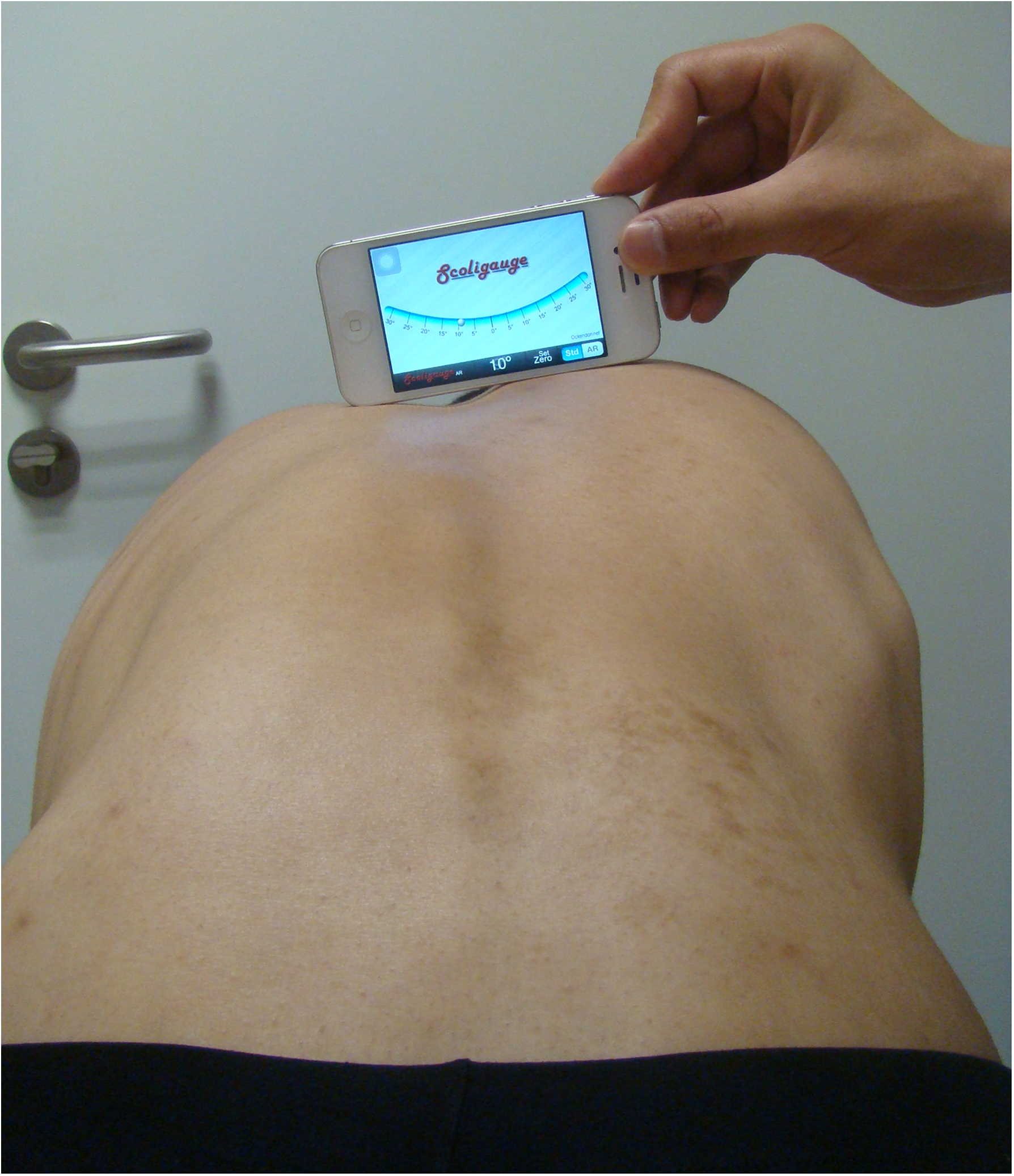

Supplement: Supplementary file 1 — Authors’ original file for figure 1 [file 12891_2014_2275_MOESM1_ESM.tif]
